# Developing an instrument to self‐evaluate the Discharge Planning of Ward Nurses

**DOI:** 10.1002/nop2.31

**Published:** 2015-09-21

**Authors:** Shima Sakai, Noriko Yamamoto‐Mitani, Yukari Takai, Hiroki Fukahori, Yasuko Ogata

**Affiliations:** ^1^ Department of Gerontological NursingSchool of NursingTokyo Women's Medical University8‐1 Kawada‐cho Shinjyuku‐wardTokyo162‐8666Japan; ^2^ Department of Adult Nursing/Palliative Care NursingDivision of Health Sciences and NursingGraduate School of Medicinethe University of Tokyo7‐3‐1 HongoBunkyo‐KuTokyo113‐0033Japan; ^3^ Department of Nursing Care ManagementGraduate School of Health Care SciencesTokyo Medical and Dental University1‐5‐45, YushimaBunkyo‐KuTokyo113‐8591Japan; ^4^ Department of Gerontological Nursing and Care System DevelopmentGraduate School of Health Care SciencesTokyo Medical and Dental University1‐5‐45, YushimaBunkyo‐KuTokyo113‐8591Japan

**Keywords:** Discharge planning, instrument development, Japan, psychometric testing, ward nurses

## Abstract

**Aims:**

To develop the Discharge Planning of Ward Nurses (DPWN), a Japanese self‐evaluation instrument for ward nurses' discharge planning practices.

**Design:**

Cross‐sectional survey.

**Methods:**

Participants were 624 ward nurses from six hospitals in Japan with a discharge planning department. Items about discharge planning practices were collected from literature and interviews with nurses and researchers. Construct validity, concurrent validity, internal consistency and test–retest reliability were tested.

**Results:**

Initially, 55 items were collected. Examination of the floor effect, item‐total, good–poor analyses and exploratory factor analysis yielded a four‐factor model with 24 items (‘teaching home‐care skills with community/hospital professionals,’ ‘identifying clients’ potential needs early in the discharge process,’ ‘introducing social resources’ and ‘identifying client/family wishes and building consensus for discharge’). The four‐factor structure was supported by confirmatory factor analysis. The DPWN correlated with scales ascertaining similar concepts, supporting concurrent validity. Internal consistency and test–retest reliability were generally satisfactory.

## Introduction

Hospital admissions increase with advancing age (Hall *et al*. 2010) and so does total healthcare expenditure, especially in Japan where the ageing of the population is among the most rapid in the world (Ministry of Health, Labour and Welfare 2011). The health insurance system was reformed in 2003 to address the spiralling costs and the healthcare system has shifted from being hospital based to community based (Ministry of Health, Labour and Welfare 2003).

As part of this shift in the healthcare system, enormous efforts have been attempted to shorten the average length of hospital stay (Sato & Fushimi 2009). As a result of this shortened length of hospital stay, older adult patients often leave the hospital with altered independence levels and vulnerable health conditions. These older adults frequently have difficulty when leaving the hospital (Buurman *et al*. 2011, Coffey & McCarthy 2013). In Japan, older clients and their families often become anxious when leaving hospital and it has been reported that some may feel resentful because they feel they were rushed out of the hospital (Koyama & Kobayashi 2012).

In this climate, careful planning is vital for smooth discharge and there are various discharge planning systems worldwide. In Japan, independent discharge planning departments with nurses and/or medical social workers support patients who are a high risk of discharge delay (Ministry of Health, Labour and Welfare 2008). The staff assist the identified patient and family decide when they leave the hospital and where they go to (e.g. long‐term care or hospitals, or home), search for long‐term care facilities/hospitals on their behalf and coordinate smooth transition in cooperation with a variety of professionals, such as nurses or social workers in the community or in facilities/hospitals (Tomura *et al*. 2011). Other countries have discharge specialists, such as nurse practitioners or clinical nurse specialists, who play a similar role (Forster *et al*. 2005, Coleman *et al*. 2006), or a discharge specialist team may be available (Sheppard *et al*. 2010).

Regardless of the various arrangements for discharge planning, ward nurses also play a key role in efficient discharge planning (Foust 2007, Suzuki *et al*. 2012). They are at the frontline of patient care and they are the ones who ascertain the needs of those high‐risk patients, take the initial action and refer them to the discharge planning department/specialist/team. However, ward nurses are still largely unfamiliar with discharge planning, especially in Japan where discharge planning is a rapidly introduced concept. Ways to help ward nurses provide more effective services for patient discharge have been explored in Japan and other countries (Ornstein *et al*. 2011, Sakai *et al*. 2011).

Several guidelines on discharge planning have been published (Lees 2010, Agency for Healthcare Research and Quality 2013), but guidelines themselves cannot be used for self‐evaluation; rather, the use of self‐evaluating instruments is to improve clinical practice (Campbell & Mackay 2001). However, the currently available self‐evaluation instruments were either prepared for discharge planning departments (Tomura *et al*. 2013) or were developed before discharge planning departments were introduced (Chiba 2005). A new self‐evaluation tool that better fits with the current discharge planning practices that guide ward nurses would be beneficial.

To date, many guidelines have been conducted in Western environments (e.g. Royal College of Nursing 2010, Agency for Healthcare Research and Quality 2013). Nurses in Japan and other countries outside Western culture might find it difficult to apply the Western idea of discharge planning in their cultural environments. For example, in Japan family ties and family involvement play a crucial role in decision‐making about health‐related issues. Caregiving is often perceived as an important familial and societal obligation, whereas in the United States greater emphasis is placed on maintaining clients’ independence and respecting their autonomy (Tanji *et al*. 2013). Client and healthcare personnel's expectations of healthcare services and assistance in discharge planning might well differ from country to country (Sentell *et al*. 2013). The new self‐evaluation instrument needs to reflect practice in Japan; the scale may also fit discharge planning in other non‐Western countries.

Based on the above literature review, this study aimed to develop an instrument for ward nurses to self‐evaluate their discharge planning practices in Japan, where many hospitals own an independent discharge planning department. It might enable ward nurses to self‐evaluate their own discharge planning practices to improve them and assess the effectiveness of any educational programmes for ward nurses. It could also be used in other countries where hospitals have a discharge department/specialist/team.

## Method

The study was conducted to develop the Discharge Planning of Ward Nurses (DPWN) scale, a Japanese instrument for ward nurses to self‐evaluate their discharge planning practices. The study consists of item development and validity and reliability testing.

### Item development

To develop the items for the DPWN, we first reviewed the literature in Japanese and international databases (i.e. PubMed, CINAHL and the Japan Medical Abstract Society database) using the keywords ‘discharge planning,’ ‘transitional care,’ ‘nursing’ and ‘cooperation.’ We also interviewed 19 registered nurses and two researchers specializing in discharge planning to obtain items on actual practices in discharge planning, asking ‘What would you do as a ward nurse to best support your clients during discharge?’ In this way, we attempted to collect a range of actual practices deemed necessary for ward nurses to give effective assistance. These nurses had more than 10 years’ ward experiences and had worked in a discharge planning department for more than 2 years. The literature review and interviews yielded 55 items for the DPWN and this list underwent statistical analyses using the survey data.

### Validity and reliability testing

#### Participants

The survey participants were all ward nurses from six acute care general hospitals in central Japan (*n* = 624). These hospitals were a convenience sample, had 300–550 beds and an average length of hospital stay of 14 days, which showed that they were typical urban, middle‐to‐large‐sized general acute care hospitals. All ward nurses at the six hospitals were contacted about possible participation in this study. Sample size was determined using the general rule for factor analytic procedure that requires a minimum of 10 respondents per item (Kline 1998).

#### Measures

The questionnaire included the following: (1) demographic and professional characteristics; (2) the 55 items for the DPWN; and (3) the Discharge Planning‐Process Evaluation Measurement (DCP‐PEM; Chiba 2005), the Oriented Problem Solving Behavior in Nursing Practice scale (OPSN; Sadahiro & Yamashita 2002) and a Visual Analogue Scale (VAS) evaluating their own discharge planning practices, all for concurrent validity testing.

The demographic and professional characteristics included age, gender, years of clinical experience, current position and educational background. The 55 items for the DPWN about self‐evaluating discharge planning began with the stem ‘How do you evaluate your practice with regard to each of the following practices in discharge planning?’ with answers recorded on a 6‐point Likert scale ranging from 1 (poor)–6 (excellent).

The following three scales were used for concurrent validity testing. The DCP‐PEM was developed in Japanese by Chiba in 2005 to self‐evaluate the discharge planning for the interdisciplinary team, not specific to ward nurses (Chiba 2005). The DCP‐PEM was used because it was the best, currently available instrument to self‐evaluate discharge planning practice; even though it was developed before discharge planning departments became common in Japan, the items included work that is now undertaken in Japan's discharge planning departments. It consisted of 26 items using a 5‐point Likert scale, with higher scores representing more effective discharge planning. Cronbach's alpha was 0·97 in this study.

The OPSN was developed by Sadahiro and Yamashita (2002) for ward nurses to self‐evaluate nursing practice in general. The OPSN was used for concurrent validity testing because it was expected that good practices in general would correspond to good discharge planning practices. The OPSN consisted of 25 items with five domains and 5‐point Likert scale and higher scores represented better practice. Cronbach's alpha was 0·97 in this study.

A VAS scale asking ‘How satisfied are you with your own discharge planning practices?’ was used because there is no gold standard to evaluate the discharge planning practices of ward nurses and it is simple, relatively easy to use and provides continuous data (De Vellis 2003, Coll *et al*. 2004). A continuous 100 mm rating scale ranging from ‘not satisfied at all’ to ‘completely satisfied’ was provided and scores were obtained by measuring the distance in millimetres from the left anchor to each participant's mark on the line.

### Data collection procedure

Researchers explained this study to the nursing managers. When they agreed to participate, the nursing managers were asked to forward questionnaires to their ward nurses. Once the ward nurses agreed to participate in this study, they completed the self‐administered questionnaires, placed their completed questionnaires in a sealed envelope and returned it to the researcher. To evaluate test–retest reliability, ward nurses from two of the six hospitals (*n *=* *195) were asked to complete the questionnaire again after an interval of 2 weeks. During the 2‐week interval, participants did not participate in any trainings, seminars or educational activities. We collected data from November–December 2011.

### Analysis

First, after examining the response distribution for extreme skewness (Tanimura *et al*. 2011), we conducted good–poor (G‐P) analysis (Hou *et al*. 2011) and the item‐total (I‐T) (Ferketich 1991) and also examined internal correlations (Ushiro 2009). In these analyses, we eliminated items with extreme skewness, non‐significance to distinguish good and poor groups, non‐significant association to the total score and high internal correlation (>0·7). We then conducted exploratory factor analysis with the maximum‐likelihood method and promax rotation to explore the underlying structure in discharge planning practice. Missing data were supplemented by the median of each item (Schaefer & Graham 2002). The number of factors was determined based on the interpretability of each factor, the number of eigenvalues greater than 1·0 and the scree plot. Items were further eliminated for parsimony when factor loadings were less than 0·45 and when communalities were less than 0·40 (Polit & Beck 2011). At this point, the final items and the subscales for the DPWN (with 24 items and a four‐factor structure) were determined.

Next, we conducted a range of validity and reliability examinations: construct and concurrent validity, internal consistency and test–retest reliability. To examine construct validity, we assessed whether the subscales could be subsumed into the second‐order, overall model of discharge planning practice using a confirmatory factor analysis. We used the goodness of fit index (GFI), comparative fit index (CFI), normed fit index (NFI) and root mean square error of approximation (RMSEA) (Kaariainen *et al*. 2011).

We examined concurrent validity by testing the associations of the subscale and total DPWN scores with the DCP‐PEM, OPSN and VAS, using Pearson's correlation coefficients. Finally, we calculated Cronbach's alphas to assess internal consistency and intra‐class correlation coefficients (ICC) for test–retest reliability (McDonald 1981). The ICC was calculated using a two‐way random effects model (Terwee *et al*. 2007). All statistical analyses were performed using SPSS version 18.0J and AMOS version 20 (IBM SPSS Japan, Tokyo, Japan).

### Ethics

Participants were informed of the purpose and methods of the study, risks and benefits of participation, confidentiality of their data and voluntary nature of participation. Written informed consent was obtained before the interviews for the item development and refinement study. The return of the anonymous questionnaire was taken as consent to participate in the main study. The study protocol conformed to the Declaration of Helsinki (as revised in Edinburgh 2000) and the study process was reviewed and approved by the ethics committee of Tokyo Women's Medical University and the administrators of the participating hospitals.

## Results

### Participant characteristics

In total, 772 questionnaires were distributed and 641 returned (response rate 83·0%). Among them, 624 questionnaires with missing data less than 5% of the total items were used (valid response rate 80·8%). Participants were on average 33·5 years old (sd 8·6), with an average of 9·3 years (sd 7·5) of nursing experience; 96% of them were female (Table 1).

**Table 1:** Participant characteristics (*N *=* *624)

	*N*	%
Mean age, years (Mean, sd)	33·5	(8·6)
Mean nursing practice duration, years (Mean, sd)	9·3	(7·5)
Gender		
Female	600	(96·2)
Male	24	(3·8)
Position		
Staff nurses	570	(91·3)
Sub nursing chiefs	37	(5·9)
Nursing chiefs	15	(2·4)
Others	2	(0·3)
Academic background		
Diploma	497	(79·7)
Associate's degree	52	(8·3)
Bachelor's degree	54	(8·7)
Graduate programme	7	(1·1)
Others	14	(2·2)

sd, Standard Deviation.

### Item selection and subscale development

Among the collected 55 items for the DPWN, no items were eliminated due to skewed distributions or through the G‐P analyses. Pearson's correlation coefficients in the I‐T analyses ranged from 0·50–0·79 except for one item that was dropped from the subsequent analyses. An additional 15 items were eliminated because of high correlations (>0·7) with other items. After these item‐level analyses, 39 items remained.

In the following exploratory factor analyses, the scree plot and parallel analysis suggested four factors and they were considered appropriate conceptually as well. Using the four‐factor model, we eliminated 11 items due to low factor loadings or communalities and further dropped four items due to content redundancy or item vagueness. The resultant final DPWN contained 24 items and the four subscales were named as follows: (1) ‘teaching home‐care skills with community/hospital professionals’; (2) ‘identifying clients’ potential needs early in the discharge process’; (3) ‘introducing social resources’; and (4) ‘identifying client/family wishes and building consensus for discharge’ (Table 2). The DPWN's mean item scores were between 2·82‐4·61 (Table 3).

**Table 2:** Final exploratory factor analysis of the DPWN: factor loadings and communality (*N* = 624)^a^

Item		Subscale 1	Subscale 2	Subscale 3	Subscale 4	Communality
Subscale 1 Providing discharge guidance in cooperation with community support team and multidisciplinary team						
I46	Teaching a patient and his/her family medical treatment consistently with staff members.	**0·877**	0·054	−0·213	0·030	0·632
I41	Working with a doctor and pharmacist to simplify the administration of intravenous drip injections and internal medicines to make it manageable for a patient and his/her family.	**0·851**	−0·001	−0·116	0·048	0·655
I43	Consulting with a nutritionist or nutrition support team (NST) about eating at home and nutrition.	**0·807**	−0·021	0·093	−0·077	0·658
I54	Preparing for home medical care (e.g. providing information about purchasing medical supplies, coordination with relevant medical institutions, etc.).	**0·755**	0·024	0·122	−0·079	0·636
I47	Checking if a patient and his/her family understand what they should do in case of an abnormal condition or emergency at home.	**0·723**	−0·048	−0·051	0·189	0·652
I42	Working with a rehabilitation staff member to teach a patient and his/her family how to perform ADL in the environment after discharge.	**0·684**	0·042	0·111	0·034	0·655
I45	Working with a discharge coordinator to arrange medical treatment that is appropriate for a patient's life.	**0·679**	−0·050	0·159	0·027	0·631
I51	Passing information about a patient's potential problems at home to the care manager, visiting doctor, visiting nurse, caregiver and public health nurse at a discharge case conference.	**0·614**	−0·046	0·198	0·050	0·606
Subscale 2 Collecting information from the client/family						
I5	Collecting information about a patient's ADL, cognition and understanding level.	0·127	**0·947**	−0·056	−0·184	0·771
I3	Collecting information about a patient's living conditions (ADL, cognitive level, dwelling environment, etc.) prior to hospitalization.	0·000	**0·822**	0·116	−0·142	0·605
I4	Collecting information about a patient's disease, progress and prognosis.	0·000	**0·674**	−0·076	0·194	0·611
I8	Collecting information about a patient's family make‐up, relationship with them and (possibly informal) key person.	−0·072	**0·586**	−0·020	0·227	0·497
I6	Collecting information about a patient's social background (life history, occupation, faith, hobbies, etc.).	−0·194	**0·529**	0·154	0·241	0·465
Subscale 3 Assisting to use social resources						
I33	Informing a patient and his/her family who can benefit from, how to apply for and what services are available through the Long‐term Care Insurance System.	0·074	0·090	**0·892**	−0·121	0·808
I34	Informing a patient and his/her family, as required, who is eligible for and how to apply for home visits by a doctor or nurse.	0·095	0·019	**0·848**	0·018	0·872
I35	Informing a patient and his/her family, as required, of the percentage of the medical payments paid by patients in the Public Assistance System.	−0·101	−0·017	**0·813**	0·072	0·621
I31	Finding out what services supporting in‐home long‐term medical care are available in the municipality where a patient resides.	0·171	−0·065	**0·539**	0·246	0·685
Subscale 4 Supporting decision‐making process						
I26	Checking if there is any difference in the future directions that a patient, his/her family and medical staff have in mind.	0·000	−0·025	0·004	**0·867**	0·730
I25	Informing a patient and his/her family of the function and role of their current hospital.	0·009	−0·078	0·151	**0·740**	0·649
I23	Informing a patient and his/her family of prospective changes in life due to the disease.	0·062	0·002	0·049	**0·736**	0·665
I14	Providing an opportunity for a patient and his/her family to be informed of their condition by the doctor according to their level of understanding.	0·056	0·190	−0·129	**0·616**	0·514
I16	Reviewing the potential problems that a patient is likely to encounter according to his/her ADL.	0·095	0·223	−0·033	**0·552**	0·582
I19	Sharing the wishes of a patient and his/her family with the doctor and discussing future directions.	0·143	0·087	0·030	**0·543**	0·533
I12	Understanding how a patient and his/her family feel about discharge from hospital and how they wish to spend their life from now on.	0·074	0·228	0·064	**0·483**	0·556

The English translation of the items has not been psychometrically tested.

a Maximum‐likelihood method with promax rotation.

DPWN, Discharge Planning of Ward Nurses.

**Table 3:** Mean item scores of the DPWN (*N = *624)

Item		Mean	sd
Subscale 1 Teaching home‐care skills with community/hospital professionals		28·67	8·16
I46	Teaching a patient and his/her family medical treatment ……..	4·06	1·09
I41	Working with a doctor and pharmacist to simplify ……………	3·72	1·23
I43	Consulting with a nutritionist or nutrition support team……….	3·40	1·28
I54	Preparing for home medical care (e.g. providing information….	3·52	1·29
I47	Checking if a patient and his/her family understand what………	3·65	1·14
I42	Working with a rehabilitation staff member to teach a patient…	3·64	1·27
I45	Working with a discharge coordinator to arrange medical……..	3·42	1·23
I51	Passing information about a patient's potential problems…….	3·27	1·42
Subscale 2 Identifying clients’ potential needs early in the discharge process		22·14	3·31
I5	Collecting information about a patient's ADL and cognition……	4·61	0·76
I3	Collecting information about a patient's living conditions………	4·45	0·86
I4	Collecting information about a patient's disease, progress and….	4·39	0·78
I8	Collecting information about a patient's family make‐up………	4·53	0·83
I6	Collecting information about a patient's social background…….	4·17	0·88
Subscale 3 Introducing social resources		12·45	4·30
I33	Informing a patient and his/her family who can benefit from……	3·21	1·26
I34	Informing a patient and his/her family, as required, who is……..	3·13	1·23
I35	Informing a patient and his/her family, as required……………..	2·82	1·17
I31	Finding out what services supporting in‐home long‐term……..	3·29	1·17
Subscale 4 Identifying client/family wishes and building consensus….		27·63	5·58
I26	Checking if there is any difference in the future directions that….	3·85	0·96
I25	Informing a patient and his/her family of the function and role….	3·67	1·06
I23	Informing a patient and his/her family of prospective changes…..	3·91	0·99
I14	Providing an opportunity for a patient and his/her family to be…..	4·13	1·02
I16	Reviewing the potential problems that a patient is likely to………	4·16	0·89
I19	Sharing the wishes of a patient and his/her family with the doctor..	3·86	1·08
I12	Understanding how a patient and his/her family feel about………	4·05	0·95
Total score		90·89	18·22

sd, standard deviation; DPWN, Discharge Planning of Ward Nurses.

### Validity and reliability testing

**Figure 1:**
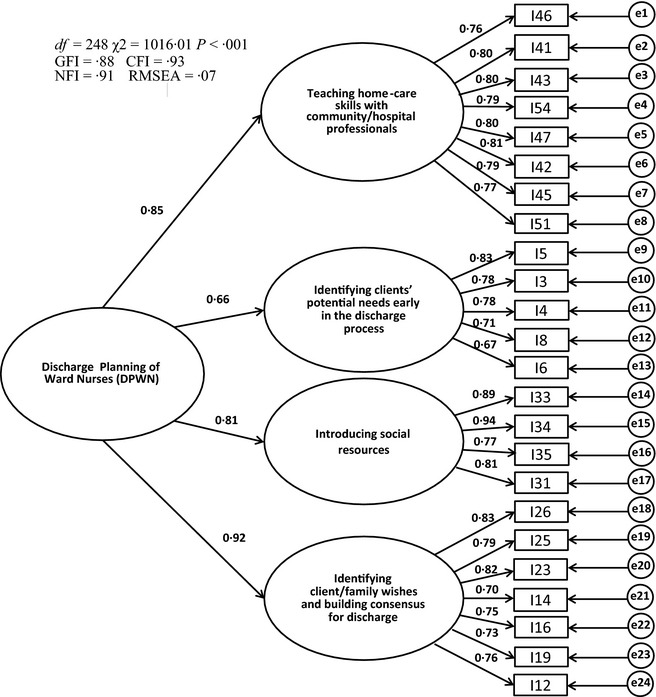
Model of the 24‐item Discharge Planning of Ward Nurses with standardized regression weights and correlations.

For construct validity testing, the confirmatory factor analysis was performed. The chi‐square value was significant (χ^2^ = 1016·01, d.f. = 248, *P *<* *0·001). The GFI, CFI, NFI and RMSEA were 0·88, 0·93, 0·91 and 0·07 respectively (Figure 1). Concurrent validity was examined by testing the associations of the DPWN scores with the DCP‐PEM, OPSN and VAS (Table 4). The subscale and total DPWN showed a statistically significant, positive correlation with the DCP‐PEM (*r *=* *0·53–0·81, *P *<* *0·001). They also showed significant correlation with the OPSN (*r *=* *0·28–0·55, *P *<* *0·001); however, the correlation was marginal for the subscale ‘introducing social resources’ (*r *=* *0·28). The subscale and total DPWN had significant correlation with the VAS (*r *=* *0·32–0·53, *P *<* *0·001); again, the correlation was marginal for the subscale ‘identifying clients’ potential needs early in the discharge process’ (*r *=* *0·32).

**Table 4:** Concurrent validity by testing the associations of Total/Subscale Scores of DPWN with DCP‐PEM, OPSN and VAS (*N = *624)

	DCP‐PEM	OPSN	Satisfaction (VAS)
DPWN (total score)	0·81^a^	0·51^a^	0·53^a^
Subscale 1: Teaching home‐care skills with community/hospital professionals	0·78^a^	0·43^a^	0·51^a^
Subscale 2: Identifying clients’ potential needs early in the discharge process	0·53^a^	0·55^a^	0·32^a^
Subscale 3: Introducing social resources	0·63^a^	0·28^a^	0·43^a^
Subscale 4: Identifying client/family wishes and building consensus for discharge	0·71^a^	0·49^a^	0·50^a^

a *P *<* *0·001, Pearson's correlation coefficient (two‐tailed).

DPWN, Discharge Planning of Ward Nurses; DCP‐PEM, Discharge Planning‐Process Evaluation Measurement; OPSN, Oriented Problem Solving Behavior in Nursing Practice; VAS, Visual Analogue Scale.

Cronbach's alpha for the total DPWN was 0·95 and those for the subscale scores ranged from 0·86–0·93 (Table 5). Test–retest reliability by ICC based on the data from 182 nurses was 0·74 (*P *<* *0·001) for the total score and ranged from 0·55–0·69 for the four subscales. The subscales ‘identifying clients’ potential needs early in the discharge process’ and ‘introducing social resources’ did not meet ICC standards (ICC > 0·64) for test–retest reliability (Yen & Lo 2002).

**Table 5:** Internal consistency (*N = *624) and test–retest reliability (*n *= 182) of the DPWN

	No. of items	Cronbach's alpha	ICC
DPWN (Total score)	24	0·95	0·74
Subscale 1: Teaching home‐care skills with community/hospital professionals	8	0·93	0·67
Subscale 2: Identifying clients’ potential needs early in the discharge process	5	0·86	0·55
Subscale 3: Introducing social resources	4	0·91	0·62
Subscale 4: Identifying client/family wishes and building consensus for discharge	7	0·91	0·69

DPWN, Discharge Planning of Ward Nurses; ICC, intra‐class correlation coefficients.

## Discussion

In this study, the four‐factor, 24‐item DPWN was developed for the self‐evaluation of ward nurses’ discharge planning practices in hospitals with discharge planning departments. The DPWN was developed based on literature review and interviews with practising nurses and researchers and its validity and reliability were examined based on data from 624 nurses. To our knowledge, this is the first attempt to a self‐evaluation instrument for ward nurses’ discharge planning practices in Japan.

### Validity and reliability

First, the DPWN's content validity was maintained according to the literature review and interviews with practising nurses regarding their actual daily discharge planning. Construct validity was examined by the confirmatory factor analysis and the chi‐square test and GFI were marginal while other indices supported the model fit. This is potentially because the chi‐square test is very sensitive and can easily reject the model (*P *<* *0·05) when the sample size is large (*n *>* *500–600) (Kaariainen *et al*. 2011); the GFI is also affected by sample size (Sharma *et al*. 2005). Given the results for the other fit indices, we considered that the four‐factor model of the DPWN showed acceptable construct validity.

Concurrent validity testing revealed that the DCP‐PEM and OPSN subscales and the VAS had moderate‐to‐high correlations with the DPWN, except for the subscale ‘introducing social resources’ and the OPSN and the subscale ‘identifying clients’ potential needs early in the discharge process’ and the VAS. The possible reason is that the OPSN focuses more on traditional inpatient care rather than the issues related to discharge planning. The DPWN might reflect Japan's contemporary healthcare situation requiring hospital ward nurses to introduce patients and their families to social resources in addition to providing individual assessment and care. The lack of strong correlation between ‘identifying clients’ potential needs early in the discharge process’ and the VAS may be because participant ward nurses have not learnt enough to consider this subscale's contents in relation to discharge planning; this suggests an area of future education for ward nurses.

Finally, Cronbach's alphas were high to adequate for both total and subscale scores. Although we removed some items, Cronbach's alpha remained high. We chose to keep some items so that the ward nurses could self‐learn from the items needed in discharge planning practice. The low ICC for the two subscales might be due to the limited number of items in these subscales. The ICC of the total score confirmed the temporal stability of the overall DPWN. Taken together, the results suggest satisfactory overall validity and reliability of the DPWN.

### DPWN components

The DPWN contains several important new items that were not used in previous instruments to self‐evaluate discharge planning practices, such as discussing anticipated health‐related lifestyle changes, sharing information with physicians about the wishes of older clients and their families and discussing future directions with clients. These items belonged to the subscale ‘identifying client/family wishes and building consensus for discharge.’ It has been suggested that ward nurses often need to assess the clients’ situations and competently assist them in the decision‐making process before the discharge planning department is involved (Watts *et al*. 2007, Naylor *et al*. 2009). The result underlined the importance of ward nurses deliberately taking time to sit with clients and their families and patiently identifying their wishes. They needed to discuss future directions together with clients until they accept the situation and truly agree with the plan. This would allow clients to leave hospital without feeling ‘kicked out.’ Thus, the new items reveal important components of the discharge planning practices of the ward nurses.

Another new key component in the DPWN is ‘introducing social resources.’ This new component also seems to be important, explaining to clients and their families how to apply for resources under the long‐term care insurance system. In Japan, 64·8% of clients in acute care hospitals are older people (Ministry of Health, Labour and Welfare 2011), who are most likely to need assistance for discharge planning. Introducing older clients and their families to social resources and reducing the burden of care are vital to help this clientele feel safe when they are home. Ward nurses are reported to have limited knowledge and understanding of the community care system (Robinson & Street 2004, Nakanishi & Morishita 2008). The DPWN should therefore help ward nurses recognize the importance of having knowledge about community care services.

Some components of the DPWN overlap with the existing literature and guidelines for discharge planning, showing that the DPWN encompasses these fundamental needs of the clients at the time of discharge: ‘identifying clients’ potential needs early in the discharge process’ and ‘teaching home‐care skills with community/hospital professionals’ (Royal College of Nursing 2010, Agency for Healthcare Research and Quality 2013). Thus, the DPWN has both unique and common for evaluating ward nurses' discharge planning practices.

### Study limitations and future research implications

This study has several limitations. First, participants were limited to nurses from the conveniently sampled six hospitals, although we made an effort to collect data from typical general acute care hospitals and nurses with various backgrounds. Future research is needed with nurses from various locations and hospital functions. Second, the DPWN was based solely on self‐evaluation; future studies should include objective evaluations by clients and families as well as third parties. Further research is also needed to confirm the DPWN's responsiveness to any interventions and its applicability in other countries.

## Conclusion

The study purpose was to develop a new instrument, the DPWN, for use in the self‐evaluation of ward nurses' discharge planning. The DPWN showed acceptable validity and reliability.

The DPWN might be used for various purposes, such as to enable ward nurses to self‐evaluate their own practices and to assess the effectiveness of educational programmes for ward nurses. Additionally, it might be useful in creating educational tools aimed at developing nurses' discharge planning competencies; moreover, new nurses may also be mentored using the tools, thus aiding the development of an educational pathway. Furthermore, assessing discharge outcomes such as patient/family satisfaction might be possible. More studies are needed to further examine the clinical utility of the DPWN both nationally and internationally.

## Implications for practice

- We developed the DPWN, which is expected to contribute to the self‐evaluation of ward nurses' discharge planning where the hospital owns the discharge planning department.
- The DPWN might serve as a self‐evaluation tool to help ward nurses improve their discharge planning and as an evaluation tool for educational programmes for ward nurses.

## Conflict of interest

No conflict of interest has been declared by the author.

## Author contributions

All authors have agreed on the final version and meet at least one of the following criteria [recommended by the ICMJE (http://www.icmje.org/recommendations/)]:

- substantial contributions to conception and design, acquisition of data, or analysis and interpretation of data;
- drafting the article or revising it critically for important intellectual content.
