# Corrigendum

**DOI:** 10.1002/nop2.49

**Published:** 2016-02-29

In Sakai *et al*. (2016) the affiliation of the first author, Shima Sakai, with the Tokyo Medical and Dental University was omitted. The full affiliation details for this paper are:

Shima Sakai^1,4^, Noriko Yamamoto‐Mitani^2^, Yukari Takai^2^, Hiroki Fukahori^3^ & Yasuko Ogata^4^

^1^Department of Gerontological Nursing, School of Nursing, Tokyo Women's Medical University, 8‐1 Kawada‐cho Shinjyuku‐ward, Tokyo 162‐8666, Japan

^2^Department of Adult Nursing/Palliative Care Nursing, Division of Health Sciences and Nursing, Graduate School of Medicine, the University of Tokyo, 7‐3‐1 Hongo, Bunkyo‐Ku, Tokyo 113‐0033, Japan

^3^Department of Nursing Care Management, Graduate School of Health Care Sciences, Tokyo Medical and Dental University, 1‐5‐45, Yushima, Bunkyo‐Ku, Tokyo 113‐8519, Japan

^4^Department of Gerontological Nursing and Care System Development, Graduate School of Health Care Sciences, Tokyo Medical and Dental University, 1‐5‐45, Yushima, Bunkyo‐Ku, Tokyo 113‐8519, Japan

We apologize for this error.
